# BRAF Mutation Status Determines the Prognostic Value of Tumor Bilaterality in Papillary Thyroid Carcinoma: A Retrospective Cohort Study

**DOI:** 10.1155/ije/1121143

**Published:** 2026-04-28

**Authors:** Shitu Chen, Fang Chen, Xingyun Su, Jie Zhou, Zhendong Chen, Zehang Xu, Bingjie Sun, Lisong Teng, Weibin Wang

**Affiliations:** ^1^ Department of Surgical Oncology, The First Affiliated Hospital, Zhejiang University School of Medicine, Hangzhou, Zhejiang, China, zju.edu.cn; ^2^ Department of Medical Oncology, The First Affiliated Hospital, Zhejiang University School of Medicine, Hangzhou, Zhejiang, China, zju.edu.cn; ^3^ Department of Pathology, The First Affiliated Hospital, Zhejiang University School of Medicine, Hangzhou, Zhejiang, China, zju.edu.cn

**Keywords:** bilaterality, BRAF mutation, papillary thyroid cancer, prognosis

## Abstract

**Background:**

While bilaterality is a frequent characteristic of papillary thyroid carcinoma (PTC), its prognostic implications in the context of BRAF genetic heterogeneity remain undefined. This study investigates the synergistic prognostic value of tumor bilaterality and BRAF mutation status in PTC recurrence risk stratification.

**Methods:**

We retrospectively reviewed 974 consecutive PTC patients who were surgically treated in the First Affiliated Hospital of Zhejiang University School of Medicine. Genomic DNA was extracted from fresh frozen tumor tissues. The BRAF mutation status was confirmed by Sanger sequencing. The cases were analyzed for various histologic and clinical parameters in order to determine the correlation between bilaterality and risk of recurrence in the different BRAF statuses.

**Results:**

Overall, bilaterality was associated with larger tumor size, gross extrathyroidal extension (ETE), and lymph node metastasis. Bilateral PTCs had a higher risk for recurrence (9.8% vs. 4.9%, *p* = 0.014) but did not retain significance after multivariable adjustment. In BRAF‐mutant patients, bilaterality was strongly associated with aggressive features such as larger tumor size, gross ETE, and lymph node metastasis and could serve as an independent risk factor for recurrence, corresponding to an HR of 2.23 (95% CI: 1.65,4.76; *p* = 0.020). BRAF‐mutant patients with bilateral tumors also exhibited a high recurrence rate of 10.3%. Notably, this effect was exclusively observed in BRAF‐mutant patients. Among these patients, the combination of bilaterality and large tumor size (> 10 mm) identified the highest‐risk subgroup, with a disease‐free survival (DFS) rate of only 86%. In contrast, these associations were not observed in BRAF wild‐type patients, regardless of tumor size or laterality.

**Conclusions:**

The current study provides initial evidence that tumor bilaterality serves as a BRAF genotype–dependent prognostic marker, revealing its synergistic effect with BRAF mutation in predicting PTC recurrence. Its prognostic value is further amplified by large tumor size, with the coexistence of bilaterality and large tumor diameter identifying a distinct high‐risk population, exclusively among BRAF‐mutant patients. Incorporating BRAF status into the assessment of patients with bilateral PTC, particularly those with larger tumors, may refine postoperative risk stratification and facilitate more tailored surveillance strategies.

## 1. Introduction

Papillary thyroid carcinoma (PTC) is the most common variant of thyroid malignancy with a rapidly increasing incidence reported worldwide [1]. In general, classic PTC is well‐behaved with a favorable prognosis; however, the mortality rate is relatively high in patients who are refractory to radioactive iodine (RAI) therapy and in those who develop tumor recurrence and distant metastasis [2, 3]. Therefore, identifying specific features that correlate with poorer outcomes in patients with PTC in efforts to improve risk stratification is critically needed in clinical practice. Clinicopathologic factors, including larger tumor size, older age, gross extrathyroidal extension (ETE), lymph node metastasis (LNM), and distant metastasis have been established as high‐risk characteristics of PTCs according to the American Thyroid Association (ATA) guidelines and the 8^th^ American Joint Committee on Cancer (AJCC) staging system [4–6].

PTC can occur as either a solitary tumor or as having two and more foci (multifocality). Bilaterality is a special subtype of multifocality with a prevalence in PTCs ranging from 13.2% to 29.8%. In addition, bilaterality is more commonly found in patients with gross ETE, advanced T stage, and LNM [7–10]. Some studies demonstrated that patients with bilateral tumors were more likely to experience recurrence [8, 11–14], while other studies have shown no difference when compared to solitary tumors [15, 16]. Our previous study, with more than 2000 PTC cases, identified bilateral disease to portend a worse prognosis, and this impact even weighed more than multifocality in predicting clinical outcomes [8]. However, it remains controversial as to whether bilaterality is an independent risk factor for recurrence in patients with PTC.

The T1799A B‐type Raf kinase (BRAF) mutation (BRAFV600E) is a main driver alteration in PTC, occurring in approximately 35%–70% of PTC patients [17]. Early studies had documented the association of BRAF mutation with malignant phenotype of PTC and cancer‐related mortality [18, 19], while others demonstrated that it was not an independent predictor of unfavorable outcomes [20]. The discrepancy in the literature may result from the different incidences of BRAF mutation among geographical regions and different tumor stages included in these study cohorts [21]. Previous studies demonstrated that the presence of BRAF mutation was correlated with multifocality and bilaterality, whereas other variables, such as tumor size, LNM, ETE, and disease‐free survival (DFS), were not significantly associated [8, 22]. It was reported that BRAF‐mutant multifocal PTC was associated with an activated Wnt signaling pathway, and the recurrence rate of these patients was as high as 20% [23, 24]. However, whether coexistent bilaterality and BRAF mutation correlate with an increased recurrence rate remains to be elucidated.

Herein, we hypothesize that bilaterality may play an important role in tumor aggressiveness with an increase in tumor recurrence based on the different genetic background of the BRAF gene. Therefore, in this study of 974 patients with a maximum follow‐up of almost 10 years, we compared the clinicopathological features and prognostic risk of bilateral PTCs with that of unilateral PTCs in wild‐type BRAF and BRAF mutation groups to test our hypothesis, which may explain the controversial clinical outcomes of bilateral PTCs for years.

## 2. Materials and Methods

### 2.1. Patients and Methods

**FIGURE 1:**
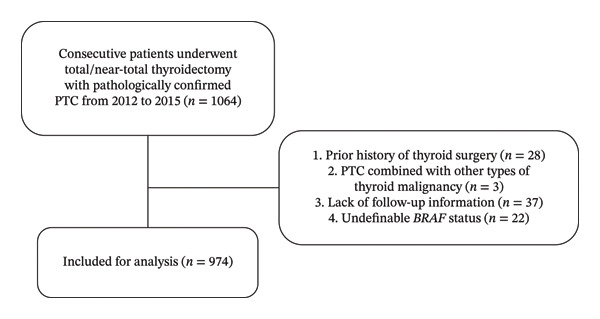
Flowchart of patients included in the study. Abbreviations: B‐type Raf kinase, BRAF; PTC, papillary thyroid carcinoma.

We retrospectively enrolled 1064 consecutive patients who underwent total/near‐total thyroidectomy and were pathologically confirmed with PTC during 2012–2015 in the First Affiliated Hospital, Zhejiang University School of Medicine (Hangzhou, China). 28 patients with a prior history of thyroid surgery, 3 patients with PTC combined with other types of thyroid malignancy, 37 patients lacking follow‐up information, and 22 patients with undefinable BRAF status were excluded. Sample size was calculated based on the primary study endpoint (disease recurrence). Expected recurrence rates (5% for unilateral PTC, 10% for bilateral PTC; hazard ratio [HR] = 2.0) were set with reference to published studies and a bilateral PTC proportion of approximately 20%–30% [8, 25]. Based on a two‐sided *α* = 0.05 and power of 0.8, the sample size was estimated using the log‐rank test formula to detect differences in recurrence risk between bilateral and unilateral PTCs. Ultimately, 974 eligible patients were included in the final analysis (Figure 1).

The surgical strategies and postoperative adjuvant treatments, including RAI treatment and thyroid‐stimulating hormone (TSH) suppression therapy for PTC during 2012–2015 in our hospital, all followed uniform standards (referring to the ATA guidelines and the Chinese guidelines for differentiated thyroid cancer [26, 27]). Prophylactic central compartment lymph node dissection was carried out following Chinese guidelines for differentiated thyroid cancer. Lateral lymph node dissection was performed for patients confirmed cytologically to have lateral LNM. All patients were initially diagnosed with PTC based on histopathology and provided written informed consent prior to surgical resection. This study was approved by the Institutional Review Board of First Affiliated Hospital, Zhejiang University, School of Medicine (2018‐381, 24 February 2018). Informed consent has been obtained from each patient after full explanation of the purpose and nature of all procedures used according to the Helsinki Declaration of 1975, as revised in 1983.

Clinicopathological data were obtained from the medical records in our hospital. Bilateral PTC was defined as cancer diagnosed on histopathology in both thyroid lobes at the same time as documented previously [8]. The pathological diagnosis was established according to WHO criteria and confirmed by two separate pathologists. Tumors were staged following the 8^th^ edition of the AJCC/TNM staging system and evaluated with the metastases, age, completeness of resection, invasion, and size (MACIS) system [5, 28].

ATA risk stratification was performed for all enrolled patients. Serum TSH, thyroglobulin (Tg) and Tg antibody measurements, neck ultrasound, and iodine‐131 whole‐body scans were carried out in order to detect disease recurrence. Disease recurrence, including local, regional and distant recurrences, was diagnosed through histologic, cytologic, radiographic, or biochemical criteria [4, 29].

Follow‐up time was defined as the time from the initial surgical treatment to the discovery of PTC recurrence or, in the case of no recurrence, to the most recent clinic follow‐up [4, 29]. The follow‐up cutoff date was December 2020. All patients were followed up every 3 months in the first year after surgery, every 6 months in the second to the fourth year, and annually from the fifth year onwards. At the end of the study period, 61 (6.3%) patients were diagnosed with tumor recurrence with a median time of 5.2 years, ranging from 4.5 to almost 10 years. Most recurrent patients were confirmed by pathological examination or CT scans, while four patients were confirmed only by Tg detectability. Among patients with structural recurrence, 55 (5.6%) patients had cervical LNM, and 2 (0.2%) patients had distant metastasis. No patient died of PTC during the follow‐up period.

### 2.2. Histological Evaluation of Tissue Specimens and DNA Extraction

Fresh frozen thyroid carcinoma tissues from 974 patients were preserved in liquid nitrogen. For DNA isolation, 25 mg of tumor tissues from microdissection was transferred to an Eppendorf tube and incubated in Protease K overnight at 56°C until the tissues were completely lysed. Genomic DNA was extracted using the QIAamp DNA Mini Kit (QIAGEN, Hilden, Germany) following the manufacturer’s recommendations. The quality and purity of DNA were assessed by a NanoDrop 1000 spectrophotometer (Thermo Fisher Scientific, Inc., Waltham, MA, USA) and evaluated by calculating the 260/280 ratio.

### 2.3. BRAF Mutation Analysis

The genomic region of BRAF Exon 15 was amplified using the following primers to obtain a 224‐bp amplicon: Forward: 5′‐TCA​TAA​TGC​TTG​CTC​TGA​TAG​GA‐3′ and Reverse:5′‐GGCCAAAATTTAATCAGTGGA‐3′. PCR reactions were performed by incubating the samples at 95°C for 10 min, followed by 38 cycles of 95°C for 30 s, 58°C for 50 s, and 72°C for 1 min. The final extension step was performed for 10 min at 72°C, and the samples were then chilled to 4°C. Sequencing of the PCR products was carried out by Sanger sequencing. The BRAF mutation was identified by comparing its sequence with the normal BRAF gene sequence in the GenBank database. In bilateral PTCs, BRAF mutation status was determined by the dominant tumor. In the current study, all BRAF mutated variants are the c.1799T > A mutation (referred to as BRAFV600E). Although this approach is controversial, McCarthy et al. and our group had previously reported that bilateral tumors in PTCs often arise from the same clonal, and tumors in both thyroid lobes usually share identical BRAF status [30, 31].

### 2.4. Statistical Analysis

The normality of continuous variables was first evaluated using the Shapiro–Wilk test. Continuous variables were presented as median ± standard deviation (SD), while categorical variables were presented as the number of cases with percentage (%). Categorical variables were compared using the Pearson’s chi‐squared test, and the Fisher’s exact test was used for case numbers ≤ 5. For multiple‐group comparison, if the overall difference was statistically significant, pairwise comparisons were conducted with Bonferroni correction for *p* values. The *t*‐test (or Wilcoxon’s rank sum test in case of no normality) was used to test the difference in the mean of the continuous variables. The survival curves were calculated by the Kaplan–Meier method, and the survival differences were compared by log‐rank tests. Cox regression multivariate analysis was conducted to identify significantly independent prognostic factors. The variance inflation factor (VIF) was used to assess multicollinearity among variables (VIF < 3 indicated no significant multicollinearity). Potential confounding variables, including patient age, sex, tumor size, gross ETE, tumor location, coexisting of Hashimoto’s thyroiditis, LNM, and RAI treatment, were adjusted. Data were analyzed by using the SPSS software Version 22.0 (SPSS Inc., Chicago, IL, USA). The threshold for statistical significance was two‐tailed *p* < 0.05.

## 3. Results

### 3.1. Baseline Characteristics

A total of 277 (28.4%) patients had bilateral PTCs, whereas 698 patients had unilateral PTCs, including 82 (8.4%) unilateral multifocal tumors and 615 (63.2%) unifocal tumors (Supporting Table 1). BRAFV600E mutation was detected in 709 (72.8%) patients, while 265 (27.2%) patients had wild‐type BRAF. 952 (97.8%) patients were in Stage I/II, while 22 (2.2%) patients were in advanced Stages III/IV. The mean MACIS score was 4.33 ± 0.92. Based on the ATA risk stratification criteria, the cohort comprised 409 (42.0%) high‐risk, 367 (37.9%) intermediate‐risk, and 198 (20.3%) low‐risk patients. The follow‐up time of this study ranged from 54 to 110 months, with a median follow‐up time of 62.3 months (95% CI: 58.1–68.1). During this period, 61 patients (6.3%) experienced structural recurrence. Among them, the median follow‐up time of patients without recurrence was 58.8 months, and the median follow‐up time of patients with recurrence was 64.2 months.

### 3.2. Effects of Bilaterality on Clinicopathologic Features and Outcomes of PTC

We initially examined the predicting effect of bilaterality on clinicopathologic characteristics of PTC (Table 1). Compared to unliteral PTCs, bilateral PTCs were more likely to present with several high‐risk characteristics, including gross ETE (23.1% vs. 16.5%, *p* = 0.016) and LNM (60.3% vs. 49.5%, *p* = 0.004). The prevalence of tumor measuring more than 10 mm was much higher in bilateral PTCs than that of unilateral PTCs (68.6% vs. 59.6%, *p* = 0.016). When MACIS system was used to evaluate the expected prognostic outcome, the score of bilateral PTCs was significantly higher (4.45 ± 0.91 vs. 4.28 ± 0.92, *p* = 0.011). Furthermore, the ATA risk stratification revealed that bilateral tumor exhibited a significantly higher proportion of high‐risk patients than unilateral cases (25.3% vs. 18.8%, *p* = 0.015), indicating poorer prognosis. Consistent with these findings, bilateral PTCs had a significantly higher risk of recurrence (9.8% vs. 4.9%, *p* = 0.014).

**TABLE 1:** Impact of bilaterality on clinicopathological features and outcomes of PTCs.

Clinicopathological characteristics	No.(%)		
	Unilateral PTCs *n* = 697	Bilateral PTCs *n* = 277	*p* value
Age ≥ 55 years	134 (19.2)	57 (20.6)	0.632
Male sex	182 (26.1)	63 (22.7)	0.20
Tumor size^a^ > 10 mm	415 (59.6)	190 (68.6)	0.016
Coexisting HT	141 (20.2)	54 (19.5)	0.796
ETE (gross)	115 (16.5)	64 (23.1)	0.016
Lymph node metastasis	345 (49.5)	167 (60.3)	0.004
AJCC III/IV stage (8^th^)	15 (2.2)	6 (2.2)	0.98
Distant metastasis	1 (0.1)	1 (0.4)	0.48
RAI therapy	260 (37.2)	118 (42.6)	0.249
ATA high‐risk	128 (18.8)	70 (25.3)	0.015
MACIS score	4.28 ± 0.92	4.45 ± 0.91	0.011
BRAFV600E	491 (70.4)	218 (78.7)	0.010
Follow‐up time, months			
Median (range)	58 (54.108)	57 (55.93)	
Tumor recurrence	34 (4.9)	27 (9.8)	0.014

Abbreviations: ATA, American Thyroid Association; BRAFV600E mutation, B‐Raf proto‐oncogene serine/threonine kinase (BRAF) valine to glutamic acid mutation at position 600; ETE, extrathyroidal extension (gross); HT, Hashimoto’s thyroiditis; MACIS, metastases, age, completeness of resection, invasion, and size; PTC, papillary thyroid cancer; RAI, radioactive iodine.

^a^Tumor size was recorded as the greatest tumor dimension.

To distinguish the significance of bilaterality from that of multifocality, we further separated 82 unilateral multifocal PTCs from total unilateral PTCs and compared their clinicopathologic characteristics with those of bilateral PTCs (Supporting Table 2). In this analysis, the bilateral group still showed significantly increased rates of gross ETE (23.1% vs. 12.2%, *p* = 0.038), higher MACIS scores (4.45 ± 0.91 vs. 4.32 ± 0.92, *p* = 0.042), and a greater prevalence of high‐risk patients (25.3% vs. 14.6%, *p* = 0.043). The prevalence of tumor measuring more than 10 mm was also much higher in the bilateral group (68.6% vs. 58.5%, *p* = 0.042).

Furthermore, we compared the 8‐year DFS rates among patients who had bilateral, unilateral‐multifocal, and unifocal PTCs. Patients with bilateral disease had the lowest survival rate of the 3 groups (88.5% vs. 93.3% and 94.4%, respectively; *p* = 0.031) (Supporting Figure 1). It was indicated bilaterality, as a specific form of multifocality, was shown to be distinct from multifocality and more strongly associated with the aggressive clinicopathologic features of PTC.

### 3.3. Association Between BRAFV600E and Clinicopathologic Features or Outcomes of PTC

Compared to wild‐type BRAF, PTCs with BRAF mutation had a significant correlation with bilaterality (30.7% vs. 21.1%, *p* = 0.009) and less with Hashimoto′s disease (16.9% vs. 28.3%, *p* < 0.001). In addition, the association was not statistically significant between BRAF mutation and other clinical characteristics (Supporting Table 3).

### 3.4. Effects of Bilaterality on Clinicopathologic Characteristics of PTCs With Respect to BRAF Status

In comparing wild‐type BRAF and BRAFV600E groups, the effects of bilaterality were notably different with respect to clinicopathologic characteristics between the two groups (Table 2). LNM rates were significantly higher in bilateral PTCs regardless of the BRAF mutation status. Bilaterality was not associated with aggressive features, such as larger tumor size and higher gross ETE rate in wild‐type BRAF patients. However, in the BRAF mutation group, the prevalence of tumor measuring more than 10 mm was much higher in bilateral PTCs than that of unilateral PTCs (67.9% vs. 59.1%, *p* = 0.026). In addition, patients with bilateral PTCs tended to have a higher risk of gross ETE (23.4% vs. 16.3%, *p* = 0.025). As for tumor stage and distant metastasis rate, there was no difference between unilateral and bilateral PTCs in the two groups. Bilateral patients also had statistically higher MACIS scores (4.55 ± 0.9 vs. 4.28 ± 0.9, *p* = 0.037), a greater proportion of high‐risk ATA cases (20.3% vs. 27.5%, *p* = 0.035), and increased recurrence rates (10.3% vs. 4.7%, *p* = 0.015), whereas the difference was insignificant in the wild‐type BRAF group. Notably, BRAF‐mutant bilateral PTCs exhibited the most aggressive prognostic profile among these four cohorts, including the highest ATA high‐risk prevalence (27.5%), MACIS scores (4.55 ± 0.9), and recurrence rates (10.3%).

**TABLE 2:** Impact of bilaterality on clinicopathological features and outcomes of PTCs with respect to BRAF status.

Clinicopathological characteristics	Wild‐type BRAF			BRAFV600E		
	No. (%) of unilateral PTCs (206)	No. (%) of bilateral PTCs (59)	*p* value	No. (%) of unilateral PTCs (491)	No. (%) of bilateral PTCs (218)	*p* value
Age ≥ 55 years	33 (16.0)	11 (18.6)	0.633	101 (20.6)	46 (21.1)	0.872
Male sex	159 (77.2)	45 (76.3)	0.883	356 (72.5)	169 (77.5)	0.160
Tumor size^a^ > 10 mm	125 (60.7)	42 (66.8)	0.320	290 (59.1)	148 (67.9)	0.026
Coexisting HT	58 (28.2)	17 (28.8)	0.921	83 (16.9)	37 (17)	0.98
ETE (gross)	35 (16.9)	13 (22.0)	0.375	80 (16.3)	51 (23.4)	0.025
Lymph node metastasis	99 (48.1)	37 (62.7)	0.047	246 (49.7)	130 (58.7)	0.019
AJCC III/IV stage (8^th^)	4 (1.9)	0	0.281	11 (2.2)	7 (2.8)	0.674
Distant metastasis	1 (0.5)	1 (1.7)	0.344	0	0	0
RAI therapy	63 (30.6)	24 (40.7)	0.29	197 (40.1)	94 (43.1)	0.509
ATA high‐risk	32 (15.3)	12 (20.4)	0.382	96 (20.3)	58 (27.5)	0.035
MACIS score	4.28 ± 0.9	4.41 ± 0.9	0.09	4.28 ± 0.9	4.55 ± 0.9	0.037
Follow‐up time, months						
Median (range)	55 (54, 96)	56 (56.93)		60 (54.108)	57 (54.91)	
Tumor recurrence	10 (4.7)	4 (6.9)	0.524	24 (4.7)	23 (10.3)	0.015

Abbreviations: ATA, American Thyroid Association; BRAFV600E mutation, B‐Raf proto‐oncogene serine/threonine kinase (BRAF) valine to glutamic acid mutation at Position 600; ETE, extrathyroidal extension (gross); HT, Hashimoto’s thyroiditis; MACIS, metastases, age, completeness of resection, invasion, and size; PTC, papillary thyroid cancer; RAI, radioactive iodine.

^a^Tumor size was recorded as the greatest tumor dimension.

### 3.5. Effects of Bilaterality on Recurrence of PTC With Respect to BRAF Status

**FIGURE 2:**
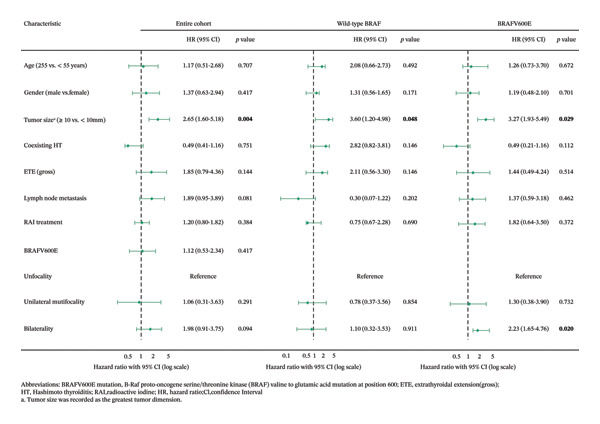
Multivariate Cox regression hazard model for recurrence in patients with PTC by BRAF status. Abbreviations: BRAFV600E mutation: B‐Raf proto‐oncogene serine/threonine kinase (BRAF) valine‐to‐glutamic acid mutation at position 600; ETE: extrathyroidal extension (gross); HT: Hashimoto’s thyroiditis; RAI: radioactive iodine; HR: hazard ratio; CI: confidence interval. Tumor size was recorded as the greatest tumor dimension.

Multivariate Cox regression analysis was conducted to identify independent prognostic factors for DFS in patients with PTCs by BRAF status (Figure 2). Regardless of the BRAF status, tumor size more than 10 mm was an independent risk factor for recurrence in these three cohorts. In BRAF‐mutant patients, however, bilaterality was another independent risk predictor for disease recurrence.

Specifically, the corresponding HR of bilaterality on DFS in the entire cohort was 2.25 (95% CI: 1.20–4.20, *p* = 0.012), but it was insignificant (HR = 1.98, 95% CI: 0.91–3.75, *p* = 0.094) after multivariable clinicopathologic adjustment (Table 3). Interestingly, in the wild‐type BRAF group, the difference between bilateral PTCs and unilateral PTCs in recurrence rate was insignificant. In contrast, in the BRAFV600E group, disease recurrence was significantly higher in patients with bilateral PTCs versus unilateral PTCs; this corresponded to an HR of 2.48 (95% CI: 1.23–5.03; *p* = 0.009), which remained significant at 2.23 (95% CI: 1.65–4.76; *p* = 0.020) after multivariable adjustment (Table 3).

**TABLE 3:** HRs of bilaterality for disease recurrence of PTCs by BRAF status.

Tumor type	Risk for tumor recurrence			
	HR (95% CI)	*p* value	Adjusted HR (95% CI)^a^	*p* value
Entire cohort	2.25 (1.20, 4.20)	0.012	1.98 (0.91, 3.75)	0.094
Wild‐type BRAF	1.36 (0.42, 4.41)	0.619	1.10 (0.32, 3.53)	0.911
BRAFV600E	2.48 (1.23, 5.03)	0.009	2.23 (1.65, 4.76)	0.020

*Note:* BRAFV600E mutation: B‐Raf proto‐oncogene serine/threonine kinase (BRAF) valine‐to‐glutamic acid mutation at position 600.

Abbreviations: HR, hazard ratio; PTC, papillary thyroid cancer.

^a^Adjusted for patient age at diagnosis, sex, tumor size, gross extrathyroidal extension, tumor location, coexistence of Hashimoto thyroiditis, and lymph node metastasis and RAI treatment.

### 3.6. Effects of Bilaterality on PTC‐Specific DFS Curves With Respect to BRAF Status

In order to study the influence of bilaterality on DFS, we performed a Kaplan–Meier analysis (Figure 3). A comparison of the curves between bilateral and unilateral PTC patients yielded a significant difference in the whole cohort (*p* = 0.024). The curve of bilateral PTC patients declined sharply, with an even more rapid decline seen in patients with BRAFV600E (*p* = 0.009). In striking contrast, the two lines remained flat without separation in BRAF wild‐type PTC patients (*p* = 0.543).

FIGURE 3Kaplan–Meier analyses of patient disease‐free survival with respect to the BRAF status. Shown are censored survival curves. The analyses were performed on the entire cohort of PTC (a), PTC patients with BRAFV600E (b), and PTC patients with wild‐type BRAF (c). BRAFV600E mutation: B‐Raf proto‐oncogene serine/threonine kinase (BRAF) valine to glutamic acid mutation at position 600; PTC: papillary thyroid cancer.

**Figure:**
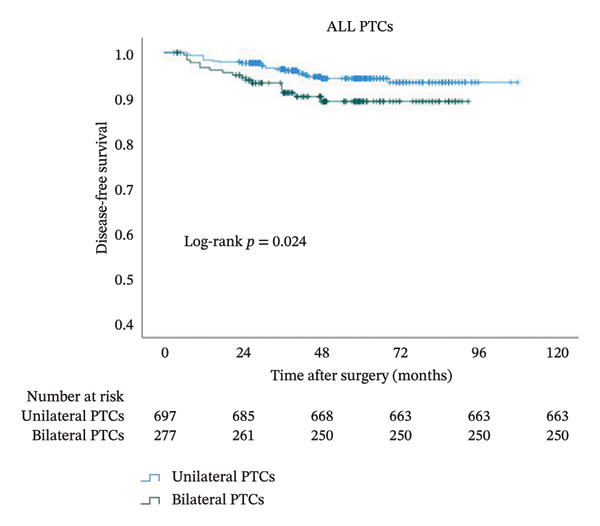
(a)

**Figure:**
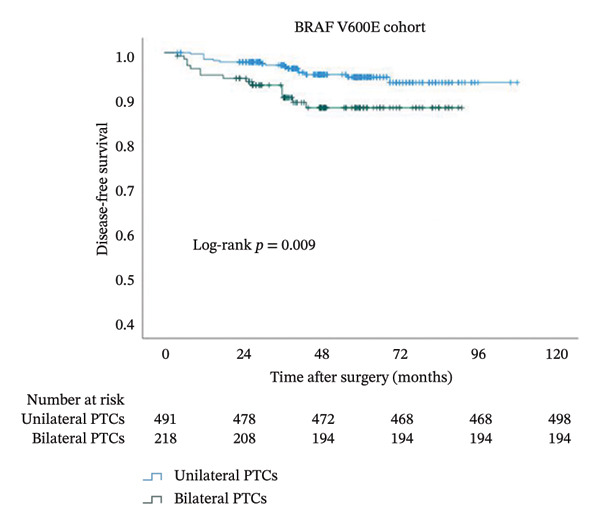
(b)

**Figure:**
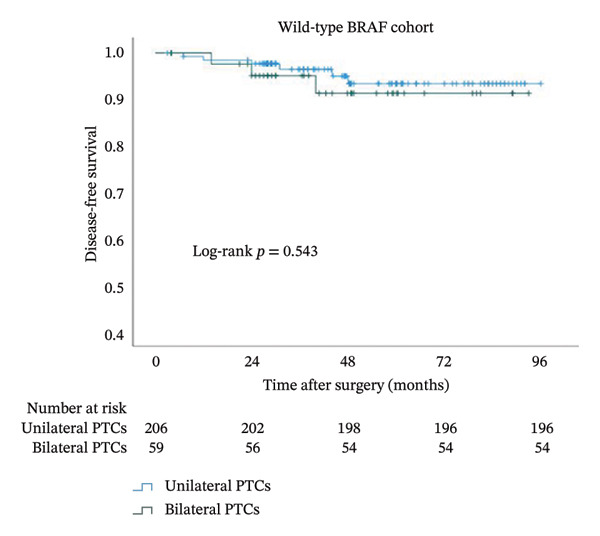
(c)

Because tumor size was identified as an independent risk factor in our multivariate logistical analysis (Figure 2) and is more frequently observed in bilateral PTCs (Table 1), we further analyzed its influence on survival together with bilaterality (Figure 4). In the overall cohort, after dividing patients into 4 groups according to bilaterality and tumor size, we observed that the DFS rate decreased dramatically from 98.5% in the unilateral tumors ≤ 10 mm group to 87% in the bilateral tumors > 10 mm (*p* < 0.001). While DFS did not differ significantly between bilateral and unilateral tumors in BRAF wild‐type patients regardless of tumor size (*p* = 0.132), a stark contrast was observed in BRAFV600E patients: the combination of bilaterality and large tumor size identified an extremely high‐risk population with a DFS rate of 86%, the lowest among all subgroups (*p* < 0.001).

FIGURE 4Kaplan–Meier analyses of 8‐year patient survival according to BRAF status. Shown are censored survival curves. The analyses were performed on the entire cohort of PTC (a), PTC patients with BRAFV600E (b), and PTC patients with wild‐type BRAF (c). Each cohort was stratified into 4 groups based on bilaterality and tumor size. Abbreviations: DFS: disease‐free survival; BRAFV600E mutation: B‐Raf proto‐oncogene serine/threonine kinase (BRAF) valine to glutamic acid mutation at position 600; PTC: papillary thyroid cancer.

**Figure:**
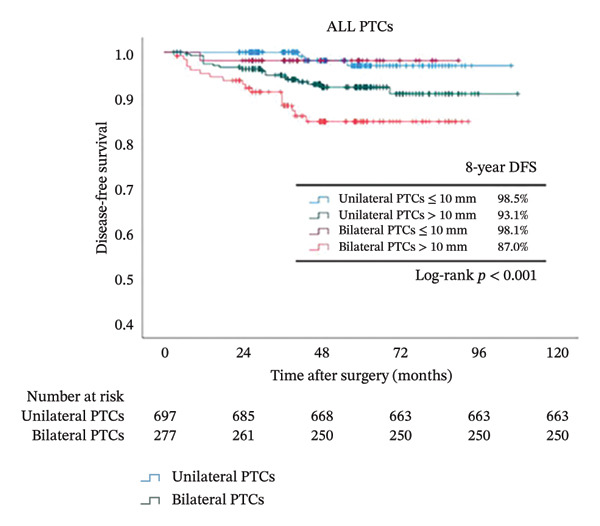
(a)

**Figure:**
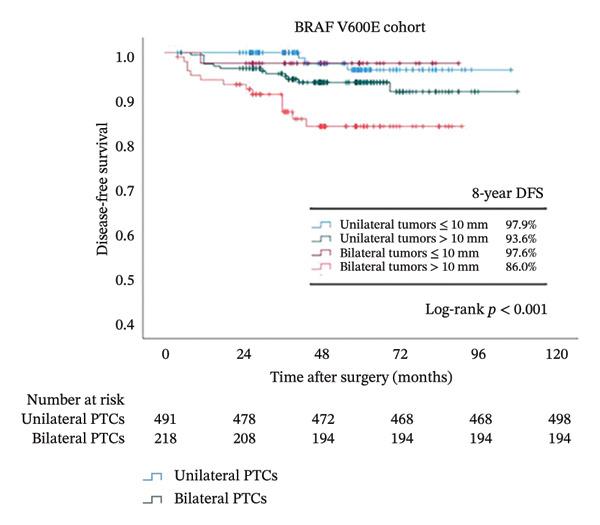
(b)

**Figure:**
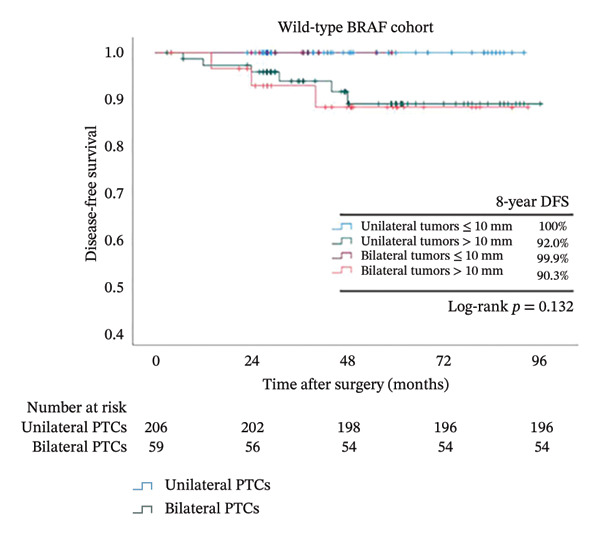
(c)

## 4. Discussion

Bilaterality is frequently observed in PTC and noted to be emerging as a risk factor for ETE and LNM [16]. Previous studies had described the increased risk of recurrence in patients with bilateral PTCs [8, 25, 31]. However, the clinical implication of bilaterality on tumor recurrence had generated different views for years [9, 15, 16]. Importantly, whether the prognostic value of bilaterality differs according to BRAF mutation status has not yet been systematically evaluated.

In the current study, regardless of BRAF status, we found a significant association between bilaterality and high‐risk clinicopathologic characteristics. Consistent with some previous studies [8, 11, 14], bilaterality had an adverse impact on DFS. Our long‐term survival analysis also indicated that bilateral PTCs had poorer clinical outcomes, although the effect was not independent. Bilaterality in PTCs tended to be a consequence of a single primary tumor with subsequent intrathyroidal metastasis to the contralateral thyroid lobe [8], which may explain why patients with bilateral tumors have an overall worse prognosis.

Consistent with the results from published literature [8, 11, 22, 32], an association of BRAF mutation and tumor bilaterality was found in the present study. A striking finding was the different effect of bilaterality on the clinical outcomes of PTC with respect to BRAF status. In BRAF‐mutant patients, tumor bilaterality was strongly associated with aggressive clinicopathological characteristics and served as an as an independent predictor of recurrence. Furthermore, the combination of bilaterality and large tumor size identified the highest‐risk population. In contrast, this association was subtle in wild‐type BRAF patients. These findings may partly explain the ambiguous role of tumor bilaterality in PTC, depending on the genetic background such as the prevalence of BRAF‐mutant PTCs in different study cohorts.

Large tumor size is a well‐established high‐risk feature in PTC, recognized by both the AJCC staging system and ATA guidelines [4–6]. In our study, larger tumors occurred significantly more frequently in bilateral PTCs and independently predicted recurrence in the overall cohort and subgroups regarding BRAF status. Importantly, among BRAF‐mutant patients, the combination of bilaterality and large tumor size (> 10 mm) identified the highest‐risk subgroup. In contrast, even the coexistence of bilaterality and large tumor size did not confer significantly additional risk in BRAF wild‐type patients. These findings suggest that larger tumor size may partly explain the poorer outcomes in BRAF‐mutant patients with bilateral PTCs, and it could amplify the prognostic impact of bilaterality specifically in the presence of BRAFV600E mutation. The synergistic interaction among BRAF mutation, bilaterality, and tumor size provides a more nuanced framework for risk stratification.

Several studies have found a significantly positive association between BRAF mutation and a more aggressive clinical course [29, 33]; however, it was not an independent predictor of worse prognosis in multivariate analysis [20, 34]. It was reported that the presence of BRAFV600E mutation had a sensitivity of 65% and a positive predictive value of only 25% in predicting the risk of recurrence [35]. Thus, the BRAF status in isolation may not substantially contribute to risk stratification in most patients [35]. Wang and Shen et al. recently demonstrated that BRAF mutation could differentiate patient age–associated and male sex–associated mortality risk of PTC, that is, BRAF‐mutant PTC patients with male sex and older age had significantly higher rates of mortality [36, 37]. In the current study, we did not succeed in proving significant associations between BRAF mutation and tumor aggressive features or prognosis. This insignificant association seems reasonable because of the relatively high prevalence of BRAF mutation (72.8%) and the rarity of recurrence (6.3%) in our study. Moreover, it had been reported in the literature that some additional genetic alterations such as NTRK gene fusions and RET/PTC rearrangements were enriched in BRAF wild‐type PTCs and were associated with LNM, ETE, and distant metastases [38, 39]. These observations align with the emerging concept that BRAF mutation functions primarily as an effect modifier rather than an independent predictor, modulating the prognostic impact of other clinicopathological factors, including bilaterality and tumor size.

Tumor location and focality could be acquired preoperatively. Multifocal papillary microcarcinoma with ETE and BRAFV600E mutation was classified as having intermediate risk for recurrence in the 2015 ATA guidelines [4]. Compared to patients with solitary tumor, patients with ipsilateral multifocality had a higher prevalence of contralateral PTC [40]. On the other hand, our previous study and some other recent studies identified bilateral disease to portend a worse prognosis, and this impact even weighed more than multifocality in predicting clinical outcomes of patients [8, 13, 25]. The 2025 ATA guideline further refined risk stratification based on tumor focality and size: T1–T3a tumors with unilateral multifocality were classified as low‐intermediate risk, with a reported recurrence rate of 10%–15%. Notably, T1–T3a tumors with bilaterality > 10 mm predicted an even higher risk of 16%–30%. However, the concurrent prognostic value of bilaterality and BRAF status has not yet been evaluated. The present study further refines this paradigm by demonstrating that this elevated risk occurs exclusively in BRAF‐mutant patients. Therefore, for BRAF‐mutant patients with bilateral tumors, particularly those with tumor size > 10 mm, an active management approach comprising timely surgery, prophylactic central neck dissection, stricter TSH suppression, and intensive follow‐up surveillance should be strongly considered.

The main limitation of our study would be its retrospective single‐institution nature, which warrants further multi‐institutional studies with long‐term follow‐up and more detailed information on treatment. Because patients have a favorable prognosis with a low recurrence rate and no disease‐related death, our follow‐up may not have been long enough to uncover the true prognostic significance of bilaterality. Second, our study did not include dynamic risk stratification data in the follow‐up period. We plan to extend the follow‐up time and collect data on dynamic risk assessment to confirm the research results described above.

## 5. Conclusions

In conclusion, this study demonstrated that the prognostic value of tumor bilaterality in PTC was determined by BRAFV600E mutation status. In BRAF‐mutant patients, bilaterality served as an independent risk factor for recurrence and, particularly when accompanied by large tumor size, defined a subgroup with markedly elevated risk. In contrast, no such association was observed in BRAF wild‐type patients. These findings suggest that bilaterality should be interpreted in the context of BRAF genetic background, and that incorporation of BRAF status into risk assessment may inform more personalized surveillance strategies for patients with bilateral PTC.

## Author Contributions

Shitu Chen: conceptualization, funding acquisition, investigation, methodology, and writing–original draft. Fang Chen: writing–review and editing. Xingyun Su: writing–review and editing. Jie Zhou: investigation and resources. Zhendong Chen: investigation and methodology. Zehang Xu: investigation and methodology. Bingjie Sun: investigation and methodology. Lisong Teng: funding acquisition, project administration, and supervision. Weibin Wang: methodology, project administration, supervision, and writing–review and editing.

## Funding

This study was supported by Grants from the National Natural Science Foundation of China (Grant nos. 82102758, 81902719, and 81972495).

## Ethics Statement

This study was approved by the Institutional Review Board of First Affiliated Hospital, Zhejiang University, School of Medicine (2018–381, 24 February 2018). Informed consent has been obtained from each patient after full explanation of the purpose and nature of all procedures used according to the Helsinki Declaration of 1975, as revised in 1983.

## Consent

Please see the Ethics Statement.

## Conflicts of Interest

The authors declare no conflicts of interest.

## Supporting Information

Supporting Table 1: Patient characteristics. Abbreviations: BRAFV600E mutation, B‐Raf proto‐oncogene serine/threonine kinase (BRAF) valine to glutamic acid mutation at position 600; ATA, American Thyroid Association; MACIS, metastases, age, completeness of resection, invasion, and size; RAI, radioactive iodine; PTC, papillary thyroid cancer; ETE, extrathyroidal extension (gross); HT, Hashimoto’s thyroiditis. a. Tumor size was recorded as the greatest tumor dimension.

Supporting Table 2: Impact of tumor location on clinicopathological features and outcomes of PTCs. Abbreviations: ATA, American Thyroid Association; MACIS, metastases, age, completeness of resection, invasion, and size; RAI, radioactive iodine; PTC, papillary thyroid cancer; ETE, extrathyroidal extension (gross); HT, Hashimoto’s thyroiditis. a. Tumor size was recorded as the greatest tumor dimension.

Supporting Table 3: Impact of BRAFV600E on clinicopathological features and outcomes of PTCs. Abbreviations: BRAFV600E mutation, B‐Raf proto‐oncogene serine/threonine kinase (BRAF) valine to glutamic acid mutation at position 600; MACIS, metastases, age, completeness of resection, invasion, and size; RAI, radioactive iodine; ETE, extrathyroidal extension (gross); HT, Hashimoto’s thyroiditis. a. Tumor size was recorded as the greatest tumor dimension.

Supporting Figure 1: Kaplan–Meier analyses of patient DFS respect to tumor location. Shown are censored survival curves. Abbreviation: DFS, disease‐free survival.

## Supporting information


**Supporting Information** Additional supporting information can be found online in the Supporting Information section.

## Data Availability

The raw data reported in this study can be accessed and downloaded from the National Genomics Data Center (NGDC) (https://ngdc.cncb.ac.cn/omix/) with accession no. OMIX007420.
