# Supplementary material for: BRAF Mutation Status Determines the Prognostic Value of Tumor Bilaterality in Papillary Thyroid Carcinoma: A Retrospective Cohort Study
Source: Int J Endocrinol. 2026 Apr 28;2026:1121143. doi: 10.1155/ije/1121143 (PMC13122560; doi:10.1155/ije/1121143)
Supplement: Supplementary file 1 — Supporting Information Additional supporting information can be found online in the Supporting Information section. [file IJE-2026-1121143-s001.zip › study protocol.docx]

Research Protocol on the Prognostic Value of Bilaterality in BRAF-Mutated Papillary Thyroid Carcinoma

Protocol Version: 1.0, November 10, 2021

1. Research Background

Papillary Thyroid Carcinoma (PTC) is the most common type of thyroid malignant tumor, with its incidence rising rapidly worldwide. It generally presents with an indolent course and favorable prognosis, yet patients unresponsive to radioactive iodine therapy have a relatively higher mortality rate, and may experience tumor recurrence and distant metastasis. Therefore, there is an urgent clinical need to identify specific features associated with poor prognosis to optimize risk stratification. According to the 2015 American Thyroid Association (ATA) guidelines, several clinicopathological factors have been identified as high-risk features for PTC recurrence, including advanced age, large tumor size, extrathyroidal extension, lymph node metastasis, and distant metastasis.

PTC can occur as a solitary tumor or with two or more lesions (multifocality). Bilaterality, a special subtype of multifocality with an incidence ranging from 13.2% to 29.8%, is more prone to extrathyroidal invasion and lymph node metastasis. Bilateral PTC has been reported to exhibit more aggressive pathological characteristics, yet the prognostic value of tumor bilaterality remains controversial to date. Our previous data and several other studies have indicated that patients with bilateral tumors have a higher risk of recurrence, with the prognostic impact even surpassing that of multifocality, while other studies suggest that bilaterality has only a modest prognostic predictive value in thyroid cancer patients. Thus, it remains controversial whether bilaterality is an independent risk factor for PTC prognosis and whether it can be universally applied to all PTC patients.

The *BRAFV600E* mutation is a major driver genetic mutation in PTC, with an incidence of approximately 35%-70%. Several studies have reported an association between the BRAF mutation and the malignant phenotype of PTC as well as tumor-related mortality, while others have suggested that the *BRAF* mutation is not an independent prognostic factor for thyroid cancer. Such inconsistencies may be attributed to the varying incidence of BRAF mutations across different geographical regions and tumor stages in the study cohorts. Our previous research and other studies have shown that the BRAF mutation is associated with multifocality, bilaterality, and a lower prevalence of concurrent Hashimoto's thyroiditis, whereas no significant correlations were found with other variables such as tumor size, lymph node (LN) metastasis, extrathyroidal extension (ETE), and disease-free survival (DFS). The recurrence rate of multifocal PTC patients with the *BRAFV600E* mutation is as high as 20%. However, the prognostic role of the combination of bilaterality and the *BRAF* mutation remains to be elucidated.

2. Research Objective

To investigate the prognostic impact of bilaterality in BRAF-mutated papillary thyroid carcinoma.

3. Research Procedures

3.1 Enrollment Criteria

A retrospective analysis was conducted on the medical records of patients diagnosed with papillary thyroid carcinoma by postoperative histopathology at the First Affiliated Hospital of Zhejiang University School of Medicine from 2012-2015. We retrospectively enrolled 1,064 consecutive patients who underwent total/near-total thyroidectomy and were pathologically confirmed with PTC during 2012-2015 in the First Affiliated Hospital, Zhejiang University School of Medicine (Hangzhou, China). 28 patients with a prior history of thyroid surgery, 3 patients with PTC combined with other types of thyroid malignancy, 37 patients lacking follow-up information, and 22 patients with undefinable BRAF status were excluded. Sample size was calculated based on the primary study endpoint (disease recurrence). Expected recurrence rates (5% for unilateral PTC, 10% for bilateral PTC; hazard ratio [HR] = 2.0) were set with reference to published studies, and a bilateral PTC proportion of approximately 20%-30%. Based on a two-sided α=0.05 and power of 0.8, the sample size was estimated using the Log-rank test formula to detect differences in recurrence risk between bilateral and unilateral PTC. Ultimately, 974 eligible patients were included in the final analysis(Figure1).

3.2 Collection of Clinical Data

The surgical strategies and postoperative adjuvant treatments including RAI treatment, and thyroid-stimulating hormone(TSH) suppression therapy for PTC during 2012-2015 in our hospital all followed uniform standards (referring to the ATA Guidelines and the Chinese guidelines for differentiated thyroid cancer. Prophylactic central compartment lymph node dissection was carried out following Chinese guidelines for differentiated thyroid cancer. Lateral lymph node dissection was performed for patients confirmed cytologically to have lateral lymph node metastasis. All patients were initially diagnosed with PTC based on histopathology and provided written informed consent prior to surgical resection. This study was approved by the Institutional Review Board of First Affiliated Hospital, Zhejiang University, School of Medicine (2018-381, 24 February 2018). Informed consent has been obtained from each patient after full explanation of the purpose and nature of all procedures used according to the Helsinki Declaration of 1975, as revised in 1983.

Clinicopathological data were obtained from the medical records in our hospital. Bilateral PTC was defined as cancer diagnosed on histopathology in both thyroid lobes at the same time as documented previously. The pathological diagnosis was established according to WHO criteria and confirmed by two separate pathologists. Tumors were staged following the 8^th^ edition of the AJCC/TNM staging system and evaluated with MACIS (metastases, age, completeness of resection, invasion, and size) system.

ATA risk stratification was performed for all enrolled patients. Serum thyroid stimulating hormone (TSH), thyroglobulin (Tg) and thyroglobulin antibody measurements, neck ultrasound, and iodine-131 whole-body scans were carried out in order to detect disease recurrence. Disease recurrence including local, regional and distant recurrences were diagnosed through histologic, cytologic, radiographic, or biochemical criteria.

Follow-up time was defined as the time from the initial surgical treatment to the discovery of PTC recurrence or, in the case of no recurrence, to the most recent clinic follow-up. The follow-up cutoff date was December 2020. All patients were followed up every 3 months in the first year after surgery, every 6 months in the second to the fourth year, and annually from the fifth year onwards. At the end of the study period, 61(6.3%) patients were diagnosed with tumor recurrence with a median time of 5.2 years, range from 4.5 to 9 years. Most recurrent patients were confirmed by pathologic examination or CT scans, while four patients were confirmed only by Tg detectability. Among patients with structural recurrence, 55(5.6%) patients had cervical lymph node metastasis and 2(0.2%) patients had distant metastasis. No patient died of PTC during the follow-up period.

3.3 Histological Evaluation of Tissue Specimens and DNA Extraction

Fresh frozen tissue samples from 1002 thyroid cancer patients were preserved in liquid nitrogen. For DNA isolation, 25 mg of microdissected tumor tissue was transferred to Eppendorf tubes and incubated overnight with proteinase K at 56°C until complete tissue lysis. Genomic DNA was extracted using the QIAamp DNA Mini Kit (Qiagen, Hilden, Germany) in accordance with the manufacturer's instructions. The quality and purity of the extracted DNA were assessed using a NanoDrop 1000 Spectrophotometer (Thermo Fisher Scientific, Waltham, Massachusetts, USA) by calculating the A260/A280 ratio.

3.4 *BRAF* Mutation Analysis

The genomic region of *BRAF* exon 15 harboring the c.1799T>A mutation was amplified using the following primers to obtain a 224 bp amplicon: forward: 5'-TCAAATGCTTGCTCTGATAGA-3', reverse: 5'-GGCCAAATTTAATCAGAGA-3'. PCR reactions were performed with an initial denaturation at 95°C for 10 minutes, followed by 38 cycles of denaturation at 95°C for 30 seconds, annealing at 58°C for 50 seconds, and extension at 72°C for 1 minute. A final extension step was carried out at 72°C for 10 minutes, after which the samples were cooled to 4°C. PCR products were sequenced by Sanger sequencing.

3.5 Data Analysis

The normality of continuous variables was first evaluated using the Shapiro-Wilk test. Continuous variables were presented as median±standard deviation (SD), while categorical variables were presented as the number of cases with percentage(%). Categorical variables were compared using the Pearson's Chi-squared test and the Fisher’s exact test was used for case number≤5. For multiple-group comparison, if the overall difference was statistically significant, pairwise comparisons were conducted with Bonferroni correction for *P*-values. The t-test (or Wilcoxon’s rank sum test in case of no normality) was used to test the difference of the mean of the continuous variables. The survival curves were calculated by Kaplan-Meier method and the survival differences were compared by Log-rank tests. Cox regression multivariate analysis was conducted to identify significantly independent prognostic factors. The variance inflation factor (VIF) was used to assess multicollinearity among variables (VIF<3 indicated no significant multicollinearity). Potential confounding variables, including patient age, sex, tumor size, gross extrathyroidal extension, tumor location, coexisting of Hashimoto thyroiditis, lymph node metastasis and RAI treatment, were adjusted. Data were analyzed by using the SPSS software version 22.0 (SPSS Inc., Chicago, IL, USA). The threshold for statistical significance was 2-tailed *P* <0.05.

4. Study Risks and Benefits; Compensation and Indemnification Measures; Subject Protection Measures Involved

This study utilizes patients' existing medical records and residual samples from the established biobank, with a minimal risk of patient privacy disclosure, which will not affect the entire clinical treatment process of patients. There may be no direct medical benefits for the participating patients themselves. We anticipate that the information obtained from this study will provide clinical guidance for patients with the same condition in the future.

Compensation: None.

Indemnification: If participants suffer study-related bodily harm or experience serious adverse events during their participation in this clinical study, they may receive corresponding indemnification in accordance with Chinese laws and regulations.

Subject Protection Measures Involved: During the study, patients' personal information such as name and gender will be anonymized with codes or numbers and strictly confidential; only the relevant attending physicians will have access to the patients’ data, ensuring full protection of privacy rights. Study results may be published in academic journals, with no disclosure of any personal identifying information.


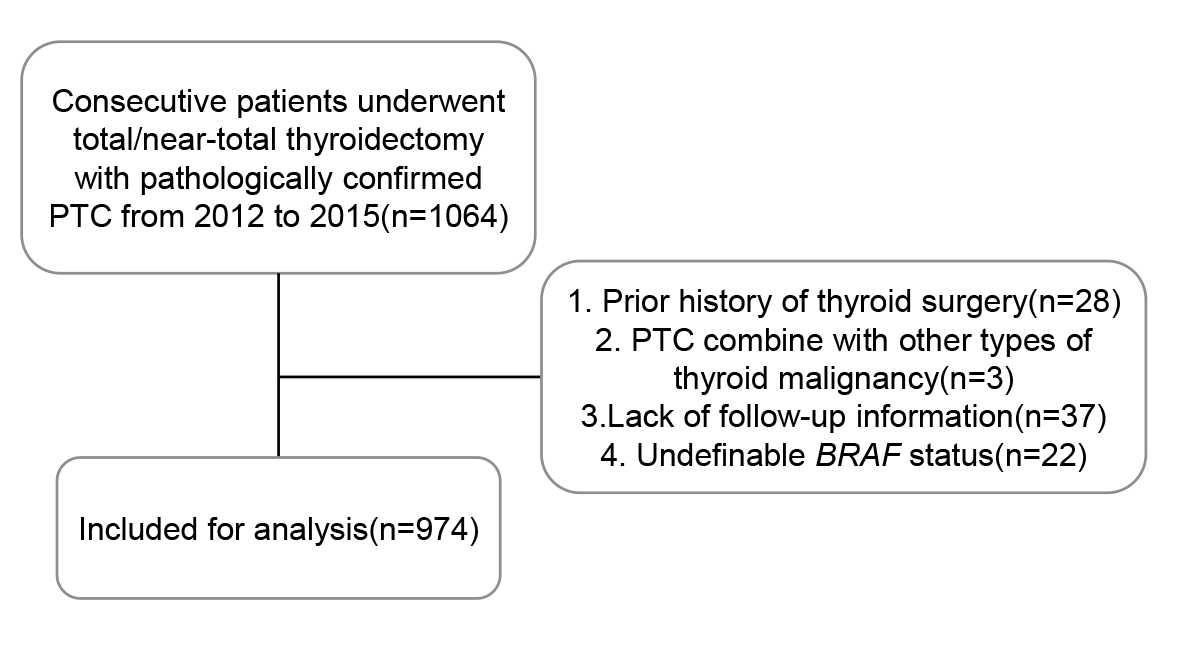


**Figure.1** Patient flowchart of patients included in the study

Abbreviations: B-type Raf kinase, *BRAF;* PTC, papillary thyroid carcinoma
