# Supplementary material for: BRAF Mutation Status Determines the Prognostic Value of Tumor Bilaterality in Papillary Thyroid Carcinoma: A Retrospective Cohort Study
Source: Int J Endocrinol. 2026 Apr 28;2026:1121143. doi: 10.1155/ije/1121143 (PMC13122560; doi:10.1155/ije/1121143)
Supplement: Supplementary file 1 — Supporting Information Additional supporting information can be found online in the Supporting Information section. [file IJE-2026-1121143-s001.zip › Supplemental meterials3rd.docx]

**Supplemental Table 1** Patient Characteristics

| Clinicopathological Characteristics | n=974, No. (%) |
| --- | --- |
| Age at diagnosis, years  <55  ≥55 | 783(80.4)  191(19.6) |
| Gender  Male  Female | 245(25.2)  729(74.8) |
| Tumor size in mm^a^  Mean ± SD  ≤10  >10 | 14.8±0.78  369(37.6)  605(62.4) |
| Coexisting HT  Yes  No | 195(20.0)  779(80.0) |
| ETE (gross)  Yes  No | 179(18.3)  795(81.7) |
| Lymph node metastasis  Yes  No | 573(58.8)  401(41.2) |
| Tumor location  Unifocality  Unilateral multifocality  Bilaterality | 615(63.2)  82(8.4)  277(28.4) |
| *BRAF* status  *BRAFV600E*  Wild-type | 709(72.8)  265(27.2) |
| AJCC stage (8^th^)  I/II  III/IV | 952(97.8)  22(2.2) |
| Distant metastasis  Yes  No | 2(0.2)  972(99.8) |
| MACIS score | 4.33±0.92 |
| ATA risk stratification  Low-risk  Intermediate-risk  High-risk | 409(42.0)  367(37.7)  198(20.3) |
| RAI therapy  Yes  No | 378(38.8)  596(61.2) |
| Follow-up time, months  Median (range)  Tumor recurrence | 62.3(54,108)  61(6.3) |

Abbreviations: *BRAFV600E* mutation, B-Raf proto-oncogene serine/threonine

kinase (*BRAF*) valine to glutamic acid mutation at position 600; ATA, American Thyroid Association; MACIS, metastases, age, completeness of resection, invasion, and size; RAI, radioactive iodine; PTC, papillary thyroid cancer; ETE, extrathyroidal extension(gross); HT, Hashimoto thyroiditis

a. Tumor size was recorded as the greatest tumor dimension.

**Supplemental Table 2** Impact of tumor location on clinicopathological features and outcomes of PTCs

| Clinicopathological Characteristics | No.(%) | | | *P-*value for | |
| --- | --- | --- | --- | --- | --- |
|  | Bilateral PTCs（n=277） | Unifocal PTCs  （n=615） | Unilateral multifocal PTCs  （n=82） | Among  three groups | Bilateral vs  Unilateral multifocal PTCs |
| Age ≥55years | 57 (20.6) | 119 (19.3) | 15 (18.3) | 0.868 | 0.982 |
| Male sex | 214 (77.3) | 458 (74.5) | 57 (69.5) | 0.343 | 0.196 |
| Tumor size^a^ >10mm | 190 (68.6) | 367 (59.6) | 48 (58.5) | 0.025 | 0.042 |
| Coexisting HT | 54 (19.5) | 129 (21.0) | 12 (14.6) | 0.389 | 0.665 |
| ETE (gross) | 64 (23.1) | 105 (17.1) | 10 (12.2) | 0.032 | 0.038 |
| Lymph node metastasis | 167 (60.3) | 302 (49.1) | 43 (52.4) | 0.008 | 0.258 |
| AJCC III/IV stage (8^th^) | 7 (2.2) | 12 (2.0) | 3 (3.6) | 0.041 | 0.701 |
| Distant metastasis | 1 (0.4) | 1（0.1） | 0 (0) | 0.79 | 0.999 |
| RAI therapy | 118(42.6) | 228(37.1） | 32(39.0) | 0.249 | 0.564 |
| ATA high-risk | 70(25.3) | 116(18.9) | 12(14.6) | 0.036 | 0.043 |
| MACIS score | 4.45±0.91 | 4.28±0.89 | 4.32±0.88 | 0.019 | 0.042 |
| Follow-up time, months  Median (range)  Tumor recurrence | 57(55,93） 27(9.8) | 59(56,108）  32 (4.9) | 58（55,108）  2（2.4） | 0.0002 | 0.036 |

Abbreviations: ATA, American Thyroid Association; MACIS, metastases, age, completeness of resection, invasion, and size; RAI, radioactive iodine; PTC, papillary thyroid cancer; ETE, extrathyroidal extension(gross); HT, Hashimoto thyroiditis

a. Tumor size was recorded as the greatest tumor dimension.

**Supplemental Table 3** Impact of *BRAFV600E* on clinicopathological features and outcomes of PTCs

| Clinicopathological Characteristics | No.(%) | | |
| --- | --- | --- | --- |
|  | Patients with wild-type *BRAF* (n=265) | Patients with *BRAFV600E*  *(*n=709) | *P*-value |
| Age≥55 years | 35 (16.6) | 146 (18.2) | 0.064 |
| Male sex | 204 (77.0) | 525 (74.0) | 0.348 |
| Tumor size ^a^ >10mm | 167 (63.0) | 439 (61.9) | 0.836 |
| Coexisting HT | 75 (28.3) | 120 (16.9) | <0.001 |
| ETE (gross) | 48 (18.1) | 131 (18.5) | 0.900 |
| Lymph node metastasis | 136 (51.3) | 372 (52.5) | 0.750 |
| AJCC III/IV stage (8^th^) | 4 (1.5) | 18 (2.5) | 0.655 |
| Bilaterality | 59 (21.1) | 218 (30.7) | 0.009 |
| Distant metastasis | 2 (0.8) | 0 (0) | 0.074 |
| RAI treatment | 88(32.4) | 290(39.8) | 0.112 |
| MACIS score | 4.33 ± 0.03 | 4.33 ± 0.05 | 0.985 |
| ATA high-risk | 44(16.7） | 154(21.6) | 0.078 |
| Follow-up time, months  Median (range)  Tumor recurrence | 55(54,96)  14 (5.1) | 58(54,108)  47 (6.4) | 0.447 |

Abbreviations: *BRAFV600E* mutation, B-Raf proto-oncogene serine/threonine

kinase (*BRAF*) valine to glutamic acid mutation at position 600; MACIS, metastases, age, completeness of resection, invasion, and size; RAI, radioactive iodine; ETE, extrathyroidal extension(gross); HT, Hashimoto thyroiditis

a. Tumor size was recorded as the greatest tumor dimension.


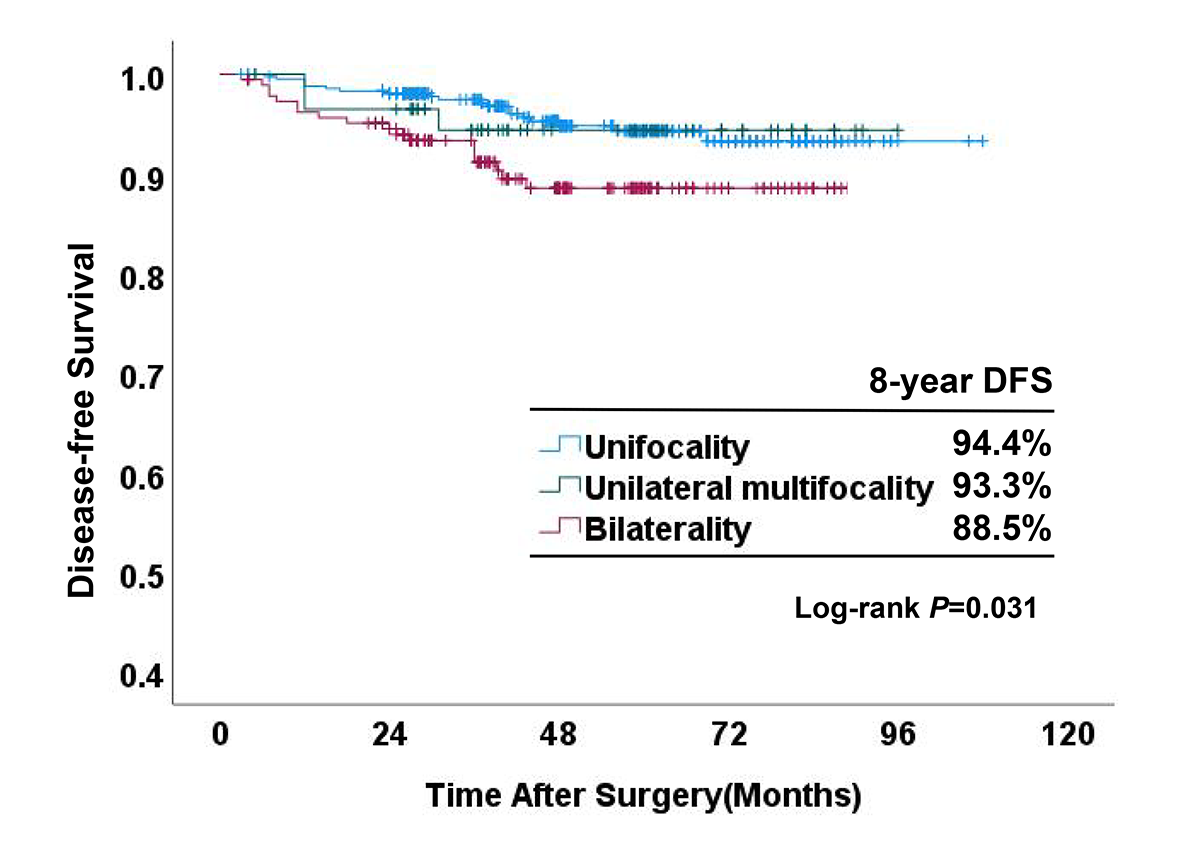


**Supplemental Fig.1** Kaplan-Meier analyses of patient disease-free survival with respect to tumor location. Shown are censored survival curves.

Abbreviations*:* DFS, disease-free survival
